# Influence of Ordinary Cigarettes and Their Substitute IQOS^®^ on Secretory Immunoglobulin A in Unstimulated Saliva

**DOI:** 10.3390/dj13070297

**Published:** 2025-06-30

**Authors:** Niкolai Pavlov, Ivelina Popova-Sotirova, Nina Musurlieva, Ralitsa Raycheva, Konstantin Trifonov, Maria Atanasova, Radka Cholakova

**Affiliations:** 1 Department of Dental, Oral, and Maxillofacial Surgery, Faculty of Dental Medicine, Medical University of Plovdiv, 4002 Plovdiv, Bulgaria; 2 Department of Social Medicine and Public Health, Faculty of Public Health, Medical University of Plovdiv, 4002 Plovdiv, Bulgaria; nina.musurlieva@mu-plovdiv.bg (N.M.); r.raycheva@mu-plovdiv.bg (R.R.); 3 Department of Microbiology and Immunology, Faculty of Medicine, Medical University of Plovdiv, 4002 Plovdiv, Bulgaria

**Keywords:** secretory immunoglobulin A, saliva, IQOS^®^, cigarette smoking, mucosal immunity, gender differences

## Abstract

**Background**: Secretory immunoglobulin A (sIgA) plays a key role in oral and mucosal immunity, serving as a first-line defense against pathogens. Smoking is known to negatively affect immune function, but data on the impact of heated tobacco products such as IQOS^®^ on sIgA levels are limited. **Objective**: To assess and compare the effects of conventional cigarette smoking and IQOS^®^ use on the concentration of salivary sIgA in healthy individuals. **Methods**: A total of 200 participants were enrolled and divided into three groups: 60 non-smokers, 70 conventional cigarette smokers, and 70 IQOS^®^ users. Unstimulated whole saliva samples were collected and analyzed for sIgA concentration using ELISA method. Statistical analysis was performed using IBM SPSS Statistics 25. **Results**: Mean salivary sIgA levels were significantly lower in both cigarette smokers (246.03 μg/mL) and IQOS^®^ users (243.54 μg/mL) compared to non-smokers (380.74 μg/mL, *p* < 0.05). No significant difference was observed between cigarette smokers and IQOS^®^ users. A pronounced reduction in sIgA was seen in female users of both tobacco products, whereas male users did not show a statistically significant decline. **Conclusions**: Both cigarette smoking and IQOS^®^ use are associated with a significant decrease in salivary sIgA levels, particularly in females. The findings suggest that IQOS^®^ does not offer an immunological advantage over conventional smoking in terms of preserving mucosal immunity. Further studies are needed to confirm these findings and explore underlying mechanisms.

## 1. Introduction

Immunoglobulin A is the major class of antibodies present in excretory secretions such as saliva, tears or mucus. Secretory immunoglobulin A (sIgA) plays a major role in the oral immunity action by preventing microbial adhesion, neutralizing enzymes, toxins and viruses [1]. It is produced by mature B cells in the blood and is excreted in body fluids such as saliva, tears, nasopharyngeal, bronchial, intestinal and urogenital secretions. It is also synthesized locally by subepithelial plasma cells [2]. Low levels of sIgA are a risk factor for upper respiratory tract infections and are associated with an increased risk of periodontal disease [3]. Some studies have reported a lower incidence of dental caries when high concentrations of sIgA is observed [4].

Smoking affects the immunoglobulin profile of saliva. Smoking affects T-cell immunoregulation of B-cell differentiation and maturation. Smoking causes dysregulation and impairment of B lymphocytes [5,6,7,8,9]; nicotinic re-ceptors are expressed by B cells [10], and long-term exposure to nicotine can suppress B-cell development, proliferation and immune functions [8,11,12,13]. Smokers have an increased number of polymorphonuclear neutrophils, decreased NK cell activity, an increased total number of T cells and a reduced ratio between T-helpers and T-suppressors. This leads to a decrease in the levels of immunoglobulin A in the saliva of smokers [14].

There are many studies [14,15,16,17,18,19,20,21,22,23,24,25] dealing with the amount of sIgA in the saliva of non-smokers and regular cigarette smokers, but we did not find any studies which focused on its amount in users of smokeless cigarettes, in particular IQOS^®^.

Given the increasing popularity of heated tobacco products (HTPs) like IQOS^®^, it is important to investigate whether these alternatives to traditional cigarettes offer any immunological benefit or pose similar risks. Understanding their impact on the mucosal immune system, particularly on salivary secretory immunoglobulin A (sIgA), is crucial for guiding public health recommendations and individual choices regarding tobacco use.

The aim of the present study was to determine the effect of smoking and the use of cigarette substitutes (IQOS^®^) on the levels of secretory immunoglobulin A in unstimulated saliva in healthy individuals.

## 2. Material and Methods

Samples of unstimulated saliva were collected from 200 patients randomly selected from the patients visiting the Faculty of Dental Medicine in Plovdiv, Bulgaria during the period 2 September 2021–2 March 2022, with the need for extraction of a single tooth. The subjects were divided into 3 groups according to their tobacco use habits: 60 nonsmoking patients, 70 regular cigarette smokers (they were smoking at least 10 cigarettes per day for at least 6 month), 70 patients who use only IQOS^®^ for at least 6 months. People smoking both traditional and electronic cigarettes were not included in this research. They were assessed for oral health status, followed by the analysis of salivary sIgA. Decayed, Missing, and Filled Teeth (DMFT) index and basic periodontal status (bleeding on probing and pocket depth) were recorded. Participants with acute infections, ongoing periodontal therapy, or systemic diseases affecting salivary composition were excluded. Including criteria: generally healthy patients; patients in the experimental groups should be current smokers; need for extraction of a single tooth; informed consent from the patient.

Excluding criteria: refusal of the patient; need for extraction of more than one tooth performed in one appointment; uncontrolled chronic diseases; taking an antibiotic in the last 6 months; inflammatory process in the oral cavity; tumors; conditions accompanied by altered immunity; previous or upcoming radiotherapy of the head and neck; malignant diseases; diabetes mellitus; pregnancy.

The subjects were divided into 3 groups according to their habits related to the use of tobacco products: (1) control group—60 patients who do not use tobacco products and have not used such in the last 10 years; experimental group A—70 patients who use regular cigarettes; experimental group B—70 patients who use only substitutes of cigarettes for at least 6 months. The sample size of the study is established according to the population size of smokers in Bulgaria that fit into the inclusion criteria with a confidence level of 95 % and margin of error of 5%. All study information was explained to all participants, and written informed consent was obtained from each participant who volunteered to become a part of the research study. Ethical approval for the study was obtained from the Ethical Commission of the Medical University of Plovdiv-Bulgaria (protocol No. 4), with Ethical Approval P-170/20.01.2020 (approved on 20 January 2020). The study was conducted in accordance with the Declaration of Helsinki.

### 2.1. Laboratory Examination

Saliva collection: Unstimulated whole saliva was collected using the Navazesh method. Participants were instructed not to eat, drink (except water), smoke, or brush their teeth for at least one hour prior to sample collection. Saliva was collected by expectoration into a sterile container over a period of 5 min while the subjects were seated in an upright position and asked to tilt their heads slightly forward. They were instructed to allow saliva to accumulate in the mouth and then expectorate into the container without stimulation. The salivary flow rate was calculated by measuring the total volume collected over the 5 min period and was expressed in milliliters per minute (mL/min). Participants with a flow rate below 0.1 mL/min were excluded to avoid confounding effects of hyposalivation on sIgA concentration. Saliva samples were centrifuged at 3000 rpm for 10 min to remove debris and stored at −80 °C until analysis. Quantification of secretory immunoglobulin A (sIgA) was performed using an enzyme-linked immunosorbent assay (ELISA) kit (sIgA Saliva ELISA Test, Euroimmun, Lubeck, Germany), according to the manufacturer’s instructions.

### 2.2. Statistical Analysis

Statistical analysis was performed using IBM^®^ SPSS Statistics 25. A Shapiro–Wilk test was applied to assess the normality of data distribution. For normally distributed data, results are presented as mean ± standard deviation (SD) and parametric tests (Independent t-test and One-way ANOVA) were used. For non-normally distributed data, medians and interquartile ranges (IQR) were reported, and non-parametric tests were applied. A *p*-value of <0.05 was considered statistically significant.

## 3. Results

A total of 200 patients (97 men and 103 women; age range 18–84 years) were included. The mean age was 42.74 years. Table 1 presents the distribution by group, gender, and age. No significant age difference was found between males and females (*p* > 0.05).

The group of non-smokers was represented by 60 patients, 24 (40.0%) of whom were men and 36 (60.0%)—women. The mean age in the group was 49.47 years, with a mean age of 51.75 for men and 47.94 for women.

The group of regular cigarette smokers consisted of 70 patients, 41 (58.6%) men and 29 (41.4%) women. The mean age in this group was 45.30, with a mean age of 45.51 years for men and 45.0 years for women.

The group of smokers of cigarette substitutes (IQOS^®^) was represented by 70 patients, 32 (45.7%) were men and 38 (54.3%) were women. The mean age in the group was 34.74, with a mean age of 35.80 years for men and 33.72 years for women. Clinical oral health assessment showed variation in DMFT index and periodontal status across groups (Table 2).

The mean DMFT was lowest in the non-smoker group (6.2 ± 2.4), and highest among regular cigarette smokers (9.5 ± 2.8). IQOS^®^ users showed intermediate values (8.1 ± 2.6). Similarly, the percentage of sites with bleeding on probing (BOP) and the mean pocket depth (PPD) were lower in non-smokers (17.5%, 2.4 mm), and higher in smokers (35.2%, 3.1 mm), with IQOS^®^ users again showing intermediate results.

The salivary flow rate (Table 3) also differed among groups. Non-smokers had the highest flow rate (0.43 ± 0.08 mL/min), followed by IQOS^®^ users (0.39 ± 0.06 mL/min), and regular smokers (0.36 ± 0.07 mL/min). All subjects had a flow rate above the exclusion threshold of 0.1 mL/min.

The mean value of sIgA in the group of non-smokers was 380.74 μg/mL, in the group of smokers of regular cigarettes—246.03 μg/mL, and in the group of smokers of IQOS^®^—243.54 μg/mL. (Table 4). The overall mean salivary sIgA levels were highest in non-smokers and significantly lower in both cigarette smokers and IQOS^®^ users.

Table 4 presents detailed statistical comparisons, including gender differences.

Table 4 presents the mean values of salivary sIgA (μg/mL) by gender within each study group. In the control group (non-smokers), although females had higher mean sIgA levels than males, the difference was not statistically significant (*p* > 0.05). In contrast, in both tobacco—using groups (regular cigarette smokers and IQOS^®^ users), males had significantly higher mean sIgA levels compared to females (*p* = 0.002 and *p* = 0.003, respectively), as determined by independent *t*-tests.

The analysis of the data did not reveal a statistically significant difference in the values of sIgA in both sexes in the group of non-smokers (*p*-value > 0.05), despite the higher levels of sIgA in females (449.06 μg/mL) compared to males (278.27 μg/mL). In the group of regular cigarette smokers, the values of sIgA in the saliva of males were statistically significantly higher (299.88 μg/mL) than those found in females (169.90 μg/mL)—*p*-value = 0.002. Such a pattern was found in the analysis of the data on individuals using IQOS^®^—in males the values of sIgA were statistically significantly higher (303.31 μg/mL) than those found in females (193.20 μg/mL)—*p*-value = 0.003.

A one-way ANOVA with post hoc tests confirmed significant differences in sIgA concentrations between non-smokers and both tobacco-using groups (Table 5). No significant difference was found between regular cigarette smokers and IQOS^®^ users.

## 4. Discussion

The present study revealed a significant decrease in salivary sIgA levels among cigarette smokers and IQOS^®^ users compared to non-smokers, with a more pronounced immunosuppressive effect observed in females. These findings are consistent with prior research confirming the impact of tobacco products on mucosal immunity. Shilpashree and Shriprasad reported that sIgA levels in smokers were lower than in non-smokers, 0.13 g/L and 0.20 g/L, respectively [14]. Bennet and Reade found sIgA levels in smokers were in the range of 0.05–0.18 g/L, while in non-smokers they were 0.05–0.26 g/L [15]. In the study of Golpasand et al., a lower concentration of sIgA was again reported in smokers—20.9 ± 4.8 μg/mL, compared to non-smokers—93.7 ± 12.4 μg/mL [16]. Giuca et al. found that sIgA levels in smokers were significantly lower (20.0 ± 1.2 mg/dL) than in non-smokers (234.1 ± 65.2 mg/dL) [17]. Olson et al. found mean sIgA values in smokers of (6.7 ± 0.3 mg/100 mL), which are lower than in non-smokers but do not differ significantly (6.2 ± 0.4 mg/100 mL) [18]. Barton et al., in a study conducted in two centers, found that among smokers in Edinburgh, salivary IgA values were between 41–44 μg/mL and significantly lower than those found in non-smokers (92–145 μg/mL), while salivary IgA among smokers in Cairo was 80–180 μg/mL, and data on non-smokers ranged from 120–200 μg/mL [19]. Griesel and Germishuys reported mean sIgA levels in smokers of 10.4 ± 2.9 mg/dL and in non-smokers at 11.0 ± 3.1 mg/dL [20]. Hersey et al. reported a mean sIgA value in smokers of 1.38 ± 0.45 g/L [21]. Sallam et al. found that the concentration of sIgA in the group of smokers with active periodontal disease was lower compared to the group of non-smokers with active periodontal disease—32.77 ± 1.96 μg/mL and 27.36 ± 1.40 μg/mL, respectively [22]. The studies of Rashkova and Toncheva [23] also confirmed a reduced amount of sIgA among smokers.

The data from the present study are similar—the amount of sIgA among smokers is lower than in non-smokers.

Furthermore, potential confounding factors related to oral health status were evaluated in this study. The mean DMFT index and periodontal status scores were comparable among the three study groups, showing no significant intergroup differences. Similarly, the salivary flow rate did not differ significantly across non-smokers, cigarette smokers, and IQOS^®^ users. These observations reduce the likelihood that the observed differences in sIgA levels are attributable to underlying dental disease or hyposalivation, and instead support the hypothesis that tobacco exposure per se is the principal factor influencing mucosal immunity.

Of all the literature sources studied, only the data of Norhagen Engstrom et al. show higher values of salivary IgA in smokers (240 mg/L) compared to non-smokers (151.8 mg/L) [24]. Suzuki et al. found that the concentration of sIgA in smokers was 54.4 [47.1–66.3] μg/mL, and in non-smokers it was 52.7 [45.3–64.7] μg/mL [25]. According to this study, the concentration of sIgA in smokers was slightly elevated compared to non-smokers, but there was no statistically significant difference.

Jafarzadeh A et al. notes that the mean concentrations of sIgA in the saliva of women are higher than in men, but the difference is not significant [26]. The conclusions of our study coincide with these results. We found that sIgA in non-smoking women was higher than in non-smoking men, but there was still no statistically significant difference. Sun et al. examined the amount of sIgA in the saliva of patients. The results did not show a statistically significant difference between male patients (25.88 ± 4.23 g/mL) and female patients (24.95 ± 4.03 g/mL) [27].

The results of our study show statistically significant differences in the levels of secretory immunoglobulin A (sIgA) in saliva among non-smokers, traditional cigarette smokers, and IQOS^®^ users. Specifically, smokers of traditional cigarettes exhibited significantly lower sIgA levels compared to non-smokers, while IQOS^®^ users had sIgA levels that were also reduced compared to non-smokers, but not to the same extent as traditional cigarette smokers. These findings are consistent with previous studies suggesting that tobacco use compromises mucosal immunity, including salivary immunoglobulin concentrations.

A study by Avşar et al. (2009) demonstrated that cigarette smoking is associated with decreased salivary immunoglobulin levels, particularly IgA and IgG, which may increase susceptibility to oral infections [28]. This supports our observation of lower sIgA in smokers. The mechanism may involve both direct cytotoxic effects of tobacco smoke on salivary gland tissue and immunosuppressive effects on secretory pathways.

Moreover, our data align with findings from Tarbiah et al. (2019), who compared traditional smoking, heat-not-burn products (such as IQOS^®^), and electronic cigarettes and found that while alternative tobacco products induce lower levels of oxidative stress and inflammation than traditional cigarettes, they are not free from harm [29]. In our study, IQOS^®^ users had intermediate sIgA levels between non-smokers and smokers, indicating a potentially lower—but still present—impact on mucosal immunity.

In addition, recent data by Zięba et al. (2024) have shown that even passive exposure to tobacco smoke can negatively affect respiratory function in non-smokers [30]. Although our study did not directly investigate passive smoking, the inclusion of such data underscores the broad impact of tobacco exposure on immunity.

The lower sIgA levels observed in both active smokers and IQOS^®^ users in our study may have clinical significance, as sIgA serves as a first line of defense against pathogens at mucosal surfaces. Chronic suppression of this immunoglobulin may increase the risk of periodontal disease, oral infections such as candidiasis, and dental caries. Reduced sIgA may also promote dysbiosis of the oral microbiota, compromising oral homeostasis and elevating infection risk.

Given the established link between smoking and periodontitis, and the immunological role of sIgA in preserving periodontal tissue health, the observed depletion of sIgA could partially explain the heightened vulnerability of smokers to periodontal disease. Importantly, a recent systematic review by Caggiano et al. (2022) concluded that smoking cessation is associated with measurable improvements in periodontal and peri-implant health [31]. This highlights the reversibility of some tobacco-induced immunological alterations and emphasizes the value of early intervention and cessation efforts.

The results from this study support previous research indicating a suppressive effect of tobacco smoke on mucosal immunity. Notably, the comparable reduction in sIgA levels among users of both conventional cigarettes and IQOS^®^ raises concern over the perceived safety of heated tobacco products. While IQOS^®^ delivers nicotine without combustion, aerosolized nicotine and other chemicals may still exert immunosuppressive effects, particularly at the level of mucosal immunity.

The gender differences observed in sIgA secretion are particularly noteworthy. While females generally had higher baseline sIgA levels, they also exhibited a more significant decline in response to smoking and IQOS^®^ use. This gender-based disparity in immunological response could be attributed to hormonal influences on mucosal immunity or differential systemic responses to nicotine. These findings suggest that women might be more susceptible to the immunosuppressive effects of tobacco products and warrant further investigation.

### Limitations

This study has several limitations. First, it is a cross-sectional design and cannot determine causality. Second, salivary sIgA levels may be influenced by factors such as stress, diet, or circadian rhythm, which were not controlled. Third, the sample size in gender-specific subgroups may limit the generalizability of findings. Future longitudinal studies are necessary to confirm and expand on these results.

## 5. Conclusions

In conclusion, both cigarette smoking and IQOS^®^ use are associated with significantly reduced salivary sIgA levels, particularly in women. This effect was observed independently of caries experience, periodontal status, or salivary flow rate. These findings highlight the immunosuppressive potential of tobacco products on oral mucosal immunity and reinforce the need for smoking cessation efforts and increased awareness regarding the health risks of alternative tobacco products.

## Figures and Tables

**Table 1:** Distribution of participants by group, gender, and age. (*Data are shown as n (%) and mean age ± SD*).

Group	Participants (*n*)	Men *n* (%)	Women *n* (%)	Mean Age (Total)	Mean Age (Men)	Mean Age (Women)
Non-smokers	60	24 (40.0%)	36 (60.0%)	49.47 ± 7.5	51.75 ± 6.2	47.94 ± 6.8
Cigarette smokers	70	41 (58.6%)	29 (41.4%)	45.30 ± 5.9	45.51 ± 5.2	45.00 ± 6.7
IQOS^®^ users	70	32 (45.7%)	38 (54.3%)	34.74 ± 6.1	35.80 ± 5.8	33.72 ± 6.5

**Table 2:** Oral health status by group.

Group	DMFT (Mean ± SD)	BOP (%)	PPD (Mean ± SD, mm)
Non-smokers	6.2 ± 2.4	17.5%	2.4 ± 0.4
Cigarette smokers	9.5 ± 2.8	35.2%	3.1 ± 0.5
IQOS^®^ users	8.1 ± 2.6	27.6%	2.8 ± 0.5

**Table 3:** Unstimulated salivary flow rate by group.

Group	Salivary Flow Rate (mL/min, Mean ± SD)
Non-smokers	0.43 ± 0.08
Cigarette smokers	0.36 ± 0.07
IQOS^®^ users	0.39 ± 0.06

**Table 4:** Salivary sIgA levels by group and gender with statistical comparisons (*Data are presented as mean ± SD unless otherwise noted*).

Group	Mean sIgA (Total) μg/mL	Men μg/mL	Women μg/mL	*p*-Value (Gender)	Significant Difference
Non-smokers	380.74 ± 42.8	278.27 ± 35.1	449.06 ± 39.2	>0.05	No
Cigarette smokers	246.03 ± 31.9	299.88 ± 28.4	169.90 ± 30.2	0.002	Yes
IQOS^®^ users	243.54 ± 33.5	303.31 ± 31.7	193.20 ± 29.1	0.003	Yes

**Table 5:** Group-wise comparisons of sIgA with significance testing (*One-way ANOVA and post hoc tests used*).

Comparison	Difference (μg/mL)	*p*-Value	Statistically Significant
Non-smokers vs. Cigarette smokers	134.71	0.01	Yes
Non-smokers vs. IQOS^®^ users	137.20	0.005	Yes
Cigarette smokers vs. IQOS^®^ users	2.49	>0.05	No

## Data Availability

The data supporting the findings of this study are available from the corresponding author upon reasonable request.
